# Time Out-of-Home and Cognitive, Physical, and Emotional Wellbeing of Older Adults: A Longitudinal Mixed Effects Model

**DOI:** 10.1371/journal.pone.0139643

**Published:** 2015-10-05

**Authors:** Johanna Petersen, Daniel Austin, Nora Mattek, Jeffrey Kaye

**Affiliations:** 1 Department of Biomedical Engineering, Oregon Health & Science University, Portland, OR, United States of America; 2 Department of Neurology, Oregon Health & Science University, Portland, OR, United States of America; Universidad Pablo de Olavide, Centro Andaluz de Biología del Desarrollo-CSIC, SPAIN

## Abstract

**Background:**

Time out-of-home has been linked with numerous health outcomes, including cognitive decline, poor physical ability and low emotional state. Comprehensive characterization of this important health metric would potentially enable objective monitoring of key health outcomes. The objective of this study is to determine the relationship between time out-of-home and cognitive status, physical ability and emotional state.

**Methods and Findings:**

Participants included 85 independent older adults, age 65–96 years (*M* = 86.36; *SD =* 6.79) who lived alone, from the Intelligent Systems for Assessing Aging Changes (ISAAC) and the ORCATECH Life Laboratory cohorts. Factors hypothesized to affect time out-of-home were assessed on three different temporal levels: yearly (cognitive status, loneliness, clinical walking speed), weekly (pain and mood) or daily (time out-of-home, in-home walking speed, weather, and season). Subject characteristics including age, race, and gender were assessed at baseline. Total daily time out-of-home in hours was assessed objectively and unobtrusively for up to one year using an in-home activity sensor platform. A longitudinal *tobit* mixed effects regression model was used to relate daily time out-of-home to cognitive status, physical ability and emotional state. More hours spend outside the home was associated with better cognitive function as assessed using the Clinical Dementia Rating (CDR) Scale, where higher scores indicate lower cognitive function (*β*_CDR_ = -1.69, p<0.001). More hours outside the home was also associated with superior physical ability (*β*_Pain_ = -0.123, p<0.001) and improved emotional state (*β*_Lonely_ = -0.046, p<0.001; *β*_Low mood_ = -0.520, p<0.001). Weather, season, and weekday also affected the daily time out-of-home.

**Conclusions:**

These results suggest that objective longitudinal monitoring of time out-of-home may enable unobtrusive assessment of cognitive, physical and emotional state. In addition, these results indicate that the factors affecting out-of-home behavior are complex, with factors such as living environment, weather and season significantly affecting time out-of-home. Studies investigating the relationship between time out-of-home and health outcomes may be optimized by taking into account the environment and life factors presented here.

## Introduction

The time an individual spends outside their residence, commonly referred to as “time out-of-home” is critical to quality of life, independence and overall health among older adults [1]. Leaving the home is necessary for participation in social and cultural activities [2], and thus closely tied to social integration and health. Indeed, depression and life satisfaction have both been linked with total time out-of-home, although only life satisfaction has been shown to have any impact on this behavior once outside the home [3]. Anecdotal studies have also reported that spending time outside the home surrounded by society helps ward off loneliness [4, 5] while quantitative studies have shown increased loneliness is associated with decreased time out-of-home [6]. However, spending time outside the home is a complex activity, requiring navigation, way finding, and physical capability [3]. Individuals with mild cognitive impairment (MCI) have been shown to not only spend less overall time out of the home [7], but also do not travel as far from the home as compared to healthy controls [8–11], perhaps due to the cognitive demands involved in leaving the home. In addition, individuals reporting higher number and frequency of physical activities also reported lower loneliness [6]. Thus, in order to leave the home one must be emotionally willing and cognitively and physically able.

Because of its correlation with emotional, physical and cognitive health, it may be possible to infer health state by objectively and continuously monitoring time out-of-home. With an appropriate technique to unobtrusively monitor time out-of-home and a complete characterization of factors affecting out of home behavior among older adults, this may enable earlier detection of important changes in cognitive status, physical ability or emotional state. Until recently, the main technique to measure out-of-home behavior was time-use surveys, where participants self-report the activities performed in and outside the home during a pre-specified interval such as an hour, day, or week [12, 13]. While this technique is useful for broad summaries of time use, it requires the detailed input of the participants which is not only subject to bias, but is impractical to report on a frequent basis for long periods of time. Similarly, life space analysis, which assesses time spent in various geospatial domains (e.g. the bedroom, the home, the neighborhood), has historically assessed one’s life space using surveys [8, 10] and self-report which encounter the same issues as time use surveys. Global positioning systems (GPS) have been proposed to objectively assess life space as they can monitor longitudinally and detect how far the individual is traveling when they leave the home [3, 9, 11]. Currently, the maximum amount of time GPS systems have been used in this context is 30 days. However, cognitive and health changes may occur on very slow time scales, and it may take years of monitoring to detect behavioral and activity changes associated with cognitive or health status. In such a long term longitudinal study, this approach may be challenging for older adults as it requires participants to remember to bring a device with them every time they leave the home, and to charge the device when they return home.

Recently, we developed a technique to continuously and unobtrusively assess the amount of time spent outside the home in older adults [6]. Using infrared motion sensors in each room of the home, and contact sensors placed on the doors to the home, we developed an algorithm that detects whether or not someone is present in the home for each five minute interval. While this technique does not gather information on distances traveled, places visited or gait outside the home, it functions without any input from or interaction by the participant. This allows the monitoring of time out-of-home for years or even decades. Because monitoring can occur over such a long time frame, it may be possible to detect changes in cognitive status, physical ability or emotional state using this system. As noted, this requires a complete characterization of not only of the interaction between time out-of-home and health, but also the association between time out-of-home and extrinsic factors known to affect behavior. For example, time out-of-home may slowly decline with both a change in cognition *and* a change in season (e.g. from summer to winter). Thus in this example, understanding and controlling for the confounding effect of change in season using a long term monitoring platform would enable more accurate inference about changes in cognition from the time out-of-home data stream.

In fact, multiple extrinsic factors may influence time out-of-home. Weather has been shown to influence depressive symptoms, affect motivation [14], and increase proclivity for headaches [15], all of which may affect time out-of-home. Also, as noted above, seasonal influences may come into play: amount of sunshine has been shown to not only influence depressive symptoms, but also affect cognition and mood—especially negative affect and tiredness [16]. Living in a retirement community may also affect time out-of-home, not only because of the proximity of resources, but also because meals may be taken on site. Individuals living in a retirement community have also been shown to take fewer steps per day [17], and those who perceive they are close to resources may be more socially active [18]. This may be tempered by socio-economic status (SES) and one’s ability to drive [19]. Each of these variables may therefore influence time out-of-home.

In this study, we use time out-of-home data from the sensor platform in addition to cognitive, emotional, and physical data from all participants to understand the relationship between cognitive function, physical ability, emotional wellbeing, and daily time out-of-home using a longitudinal *tobit* regression model. We had three hypotheses regarding the association between time out-of-home and health: (i) individuals with better cognitive status will spend more time outside the home, (ii) individuals with less pain and better physical health will spend more time outside the home, and (iii) individuals in a more positive emotional state (less loneliness and less low mood) will spend more time outside their home. We also describe several contextual variables including weather, season and living environment, and describe their relationship to time out-of-home. This characterization of variables affecting daily time out-of-home may enable continuous monitoring of cognitive health, emotional wellbeing, and physical function of older adults using objective assessments of time out-of-home.

## Materials and Methods

### Participants

The participants and data for this study come from the ISAAC [20] and ORCATECH Life Laboratory cohorts, two ongoing observational studies aiming to understand the complex relationship between daily behavior and health outcomes. The minimum age for participation in the ORCATECH Life Laboratory is 65 years, and that for the ISAAC cohort is 80 years (or 70 years for non-whites). Participant inclusion criteria for both cohorts include living independently in an apartment or house larger than a studio apartment, a minimum score of 25 on the Mini-Mental State Examination [21], and a maximum score of 0.5 on the Clinical Dementia Rating [22] scale. Participants were also required to be in average health for their age. All participants provided written informed consent before participating in study activities. These protocols were approved by the Oregon Health & Science University Institutional Review Board (IRB #2765 and #2353). Recruitment into these ongoing observational studies began in 2007, and at the time this study started (December of 2011), these two cohorts comprised 149 seniors in the Portland community who had been monitored for the past 3–5 years. Of these participants, 85 were living alone independently at the time of this study and were included in this analysis. Participants who lived with other people (*n* = 48) were excluded as the out-of-home algorithm has only been validated for individuals living alone. In addition, periods where participants had overnight visitors were excluded from the analysis as the algorithm has not been validated in such multi-person situations. These were taken into account via a weekly on-line questionnaire (described below). Participants who lived in advanced care facilities (*n* = 16) were also excluded as regular nurse visits, especially those while the subject is away from home, are known to bias the estimate of time out-of-home.

### Data and Measures

The home-based assessment system and protocol has been described in detail elsewhere [20]. Briefly, all participants received a core set of technologies in their home including pyroelectric infrared motion sensors (MS16A, x10.com) in each room, contact sensors (DA10A, x10.com) on the refrigerator and doors to the home, and a personal computer (they could also opt to use their own). A line of motion sensors was also placed in a straight line in a frequently used walkway, and used to assess in-home walking speed (discussed in more detail below). Participants also received an online health questionnaire to complete each week that asks questions about events that may affect activity patterns (as measured using the in-home sensors), including changes in health. In addition, each participant is assessed in-home at baseline and annually using a standardized battery of tests consisting of several physical and neuropsychological instruments [20].

In June of 2012, all participants were administered an online version of the 20-item UCLA Loneliness Scale [23], a validated survey for assessing loneliness in seniors [24]. Despite the fact that we have been monitoring subjects for multiple years, we limited the scope of this study to the year surrounding the single administration of the UCLA Loneliness Scale (December 6, 2011 through December 4, 2012) because we hypothesized that loneliness levels would affect the daily time out-of-home.

All variables (except the Loneliness Scale score and style of living area) used in this study came from one of four sources: the yearly physical and neuropsychological examinations (collected in the home by a clinician), the weekly health form (collected weekly online by self-report of the participant), the sensor platform data (collected continuously, unobtrusively and objectively in the home), or from publicly available resources online (daily weather and seasonal data only). This section details each variable used in the model. The variable name that is used in the results table is italicized for ease of reference.

#### Time out-of-home

*Time out-of-home* (the outcome variable) was computed probabilistically using a logistic regression classifier and several features of the in-home data that correspond to the presence of an individual in the home [6]. This technique has high sensitivity (94%) and specificity (98%), and is completely unobtrusive. Days where the subject was away from home overnight (e.g. due to hospital stay or vacation) were excluded from the analysis.

#### Cognitive status

Cognitive function was assessed annually in the home by a clinician using The Clinical Dementia Rating (*CDR*) Scale, a 5-point scale used to characterize each participant’s level of cognitive impairment [22]. A rating of 0.5 on this scale indicates mild cognitive impairment. As noted, clinical exams were given at baseline and annually for each participant. For each day in the present study, the data from the most recent clinical evaluation was used for all clinical variables.

#### Physical ability

*Pain level* was assessed weekly using the online health form. Participants were asked to rate their pain by indicating the number that best described their pain on average in the last week, which they answered on an 11-point Likert scale (0–10) with lower scores indicating less pain. Participants failed to complete the weekly health form a median of 7.6 weeks during the monitoring period. On the weeks they completed the weekly health form, their pain responses were often consistent: participants reported the same level of pain on 47% of consecutive weeks. Thus we performed local linear interpolation on pain values for those days that were missing the self-report of pain.

*Clinical walking speed*, an important aspect of physical function, was assessed annually using a timed walk [25]. Daily in-home walking speed was also assessed unobtrusively using an array of sensors in the home (described next, below). As noted, clinical exams were given at baseline and annually for each participant; the data from the most recent clinical evaluation was used for this clinical variable.

*Normalized daily walking speed* was calculated using a line of four motion sensors positioned in series on the ceiling. The field of view of the sensors was restricted so they fired only when the participant passed directly underneath them. The distance between sensors was recorded to allow adequate calculation of velocity as the participant passed through the line of sensors [26]. While the data from these sensors is highly correlated with true walking speed, the variability in sensor placement and refractory period means there is typically a constant offset between true walking speed and calculated walking speed for each line of sensors. As a result, the raw walking speed is typically not comparable cross-sectionally because the constant offset will differ across individuals. To account for this, we normalized the measures of walking speed for each individual by the median and IQR for that participant [26]. Then, because the participant can walk through the line multiple times in a day, the median normalized walking speed was determined for each day and used as normalized daily walking speed in the model. Thus, this variable represents the deviation in daily walking speed from an individual’s median.

#### Emotional state

As noted, *loneliness* was assessed in June of 2012 using the 20-item UCLA Loneliness survey. This survey asks questions such as “I do not feel alone” where response options are: (1) Never, (2) Rarely, (3) Sometimes and (4) Often. The loneliness survey was administered to participants in conjunction with their regular weekly health form which is programmed to appear on participant computers every time they log in to the computer until it is completed each week. This format elicits superior response rates as it avoids the need for participants to regularly check email, but still requires participants to regularly use the computer. The loneliness survey appeared immediately after completion of the weekly health form. Participants were told the general nature of the ensuing questions and given the option to accept, decline, or postpone survey completion. If a participant opted to postpone survey completion, it appeared after subsequent weekly health forms until either the subject completed the entire survey, declined to complete the survey, or a period of two months passed. Because this survey was administered online rather than in-person, we also added a response for ‘do not wish to answer’ so participants would not be forced to answer questions that made them uncomfortable.

After reversing the value of negative questions, the value of each answer was summed across the twenty questions to give a loneliness score ranging from 20 to 80, with 80 being the loneliest. Surveys where an individual marked ‘do not wish to answer’ on any question were treated as missing data because the sum score could not be determined. In order to include those individuals who either marked ‘do not wish to answer’ on at least one question or did not complete the survey, we included an indicator variable for those who did not complete the Loneliness Scale, and set their loneliness score to zero. This method for handling missing data is known to be biased when the data is missing at random; however in this case, the data is likely not missing at random. To obtain non-biased coefficients, it is necessary to explicitly model the missing data mechanism. As shown in Table 1, participants who did not complete the survey were more likely to be cognitively impaired because completion of the survey required regular computer use which has been linked with cognitive ability [27]. To account for this missing data mechanism, we included a binary indicator for those who did not complete the survey and had a CDR score of 0.5. Further, coefficients from this model were similar to those obtained if all subjects with missing loneliness scores were dropped from the model altogether, indicating an adequate missing data model. Participants who completed the survey were younger than those who did not, but were the same on all other demographic variables (Table 1).

**Table 1:** Demographic characteristics of study population.

Characteristic	Missing/Incomplete Loneliness Score (*n* = 43)	Known Loneliness Score (*n* = 42)	Total Cohort (*n* = 85)	p-Value
Age (years)				0.009
Mean (SD)	87.96 (5.85)	84.68 (7.21)	86.36 (6.79)	
Gender				0.778
Male	6	5	11	
Female	37	37	74	
Race				0.469
White	34	37	71	
Black	6	4	10	
Asian	3	1	4	
Education				0.652
Some High School	1	1	2	
High School Graduate	9	5	14	
Partial College	12	17	29	
College Graduate	11	12	23	
Graduate Degree	10	7	17	
Living Environment				0.208
Retirement Community	33	27	60	
Independent Housing	10	15	25	
CDR Score				0.048
0	35	40	75	
0.5	8	2	10	

*Low mood* was assessed weekly via a binary question on the online health form that asked “Have you felt downhearted or blue for three or more days in the past week?” Because the question was not specific to particular days the participant felt blue, each of the seven days up to and including a positive answer to this question was coded with a dummy variable indicating ‘low mood’. As noted, participants failed to complete the weekly health form a median of 7.6 weeks during the monitoring period, corresponding to a median of 21% missing data per subject. Because low mood may be associated with not logging into the computer to complete the weekly health form, we included a dummy variable in the model to control for days where the participant had not completed the corresponding weekly health form. In this way, the model can fit the data separately for individuals with missing ‘low mood’ data.

#### Contextual variables

Demographic data was collected for each participant at the first in-home clinical evaluation. In this study, we included *sex*, *race*, *gender*, years of *education*, *age* (taken as age in years on the first day of this study), and style of living area (*retirement community* or residential) in the model as these were considered likely to affect time out-of-home (we did not include socio-economic status as it was highly correlated with education in our cohort).

*Season* was included as the hour the sun sets each day in Portland, OR, USA. This data was downloaded from the Astronomical Applications Department of the US Navy, a publically available resource [28]. One hour was added to the sunset time for days during daylight savings time. Amount of *precipitation*, *max temperature*, and *wind speed* were downloaded as part of a global summary of the day for the Portland metropolitan area from the National Climatic Data Center (Portland International Airport, Station number 72698024229), a publically available online weather database [29]. *Weekday* was also included in the model.

### Data Analysis

**Fig 1:**
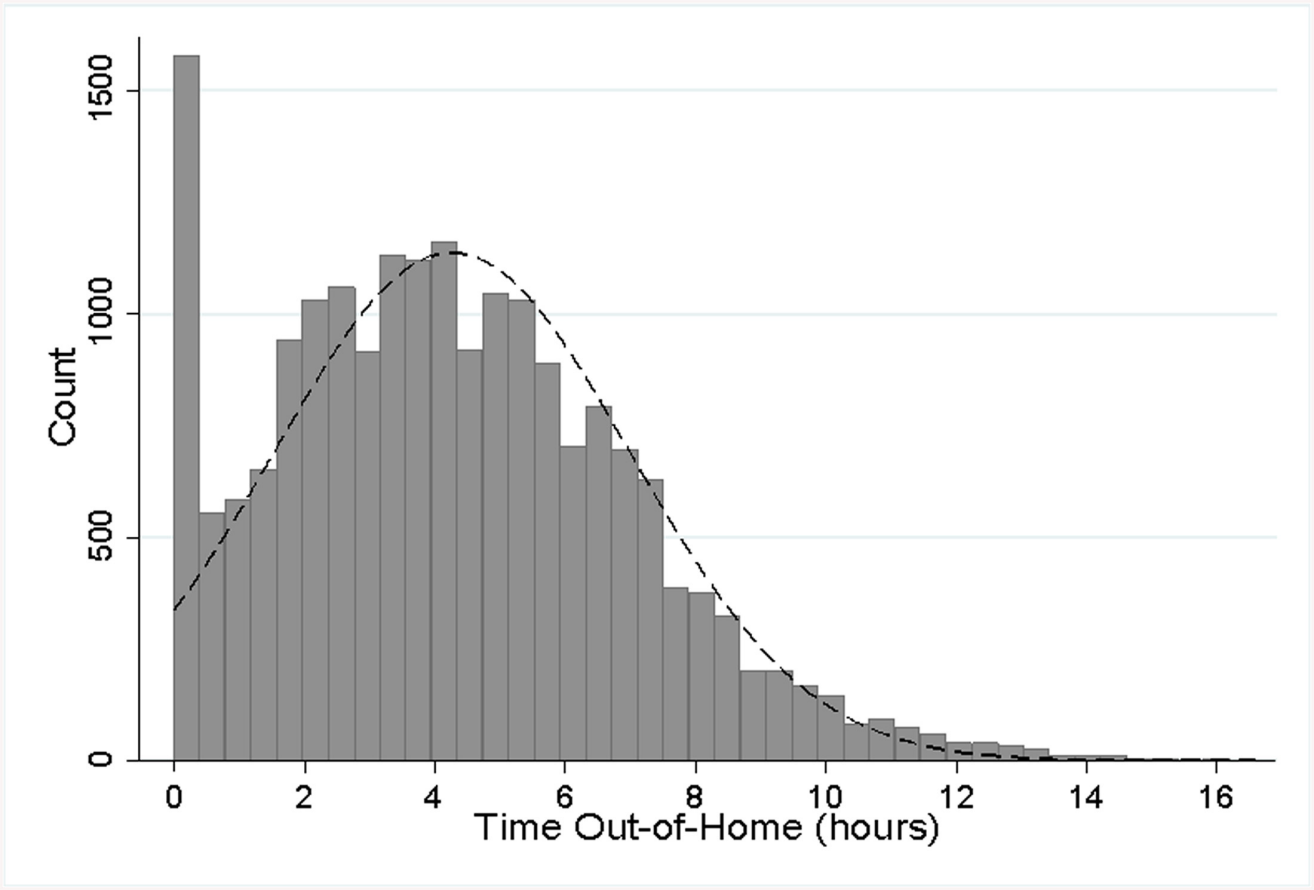
Histogram of the daily hours spent outside the home, showing the limit at zero. A normal distribution curve is plotted as a dashed line to show the data is approximately normally distributed except at and below zero.

A histogram of the daily time out-of-home data is shown in Fig 1. The data is approximately normally distributed over values greater than zero, but there is an accumulation of values around zero. This accumulation around zero results in a bimodal data pattern characteristic of censored variables, and makes it unreasonable to treat the data as a normally distributed conditional random variable. The *tobit* model directly accounts for the accumulation at zero, and has been validated as a superior approach to analyzing such censored and corner solution outcome variables [30, 31]. We therefore analyzed the relationship between total daily time out-of-home and cognitive status, physical ability and emotional state using a longitudinal *tobit* panel model in STATA.

It is important to emphasize that the *tobit* model has a slightly different interpretation than the more commonly used ordinary least-squares (OLS) regression model (not used here), which is especially informative for this application. First, we note again that the time out-of-home data is bimodal, with an accumulation of values at zero representing no time out-of-home for that day, and a normal distribution above zero. However, only a single *β* coefficient is estimated for each independent variable in the *tobit* model, which contains information about these two types of cases: those with zero time out-of-home and those with nonzero time out-of-home. To correctly interpret the effect of each independent variable on time out-of-home, we first calculated the contribution of the two cases (zero time out-of-home and non-zero time out-of-home) to the *β* coefficients calculated by the *tobit* model using a technique described by Roncek [32]. The contribution of *β* on the zero values describes the percent increase in probability that the participant will leave the home and is represented as P(out). The effect of *β* on the nonzero values describes the change in time out-of-home given the participant left the home that day and will represented as T_out_|Out. The second interpretation is closer to that provided by OLS, while the first is closer to the odds ratio provided by a logistic regression.

In order to ensure coefficient estimates were not biased by multicollinearity, the variance inflation factor (VIF), a standard diagnostic tool for multicollinearity, was calculated for each independent variable included in the model. The VIF for all variables in the model was below 2.5, indicating any multicollinearity can safely be ignored [33]. Confidence intervals for the parameter estimates were determined using 1000-fold bootstrap replicates. A *p-*value of 0.05 was considered significant.

## Results

In this section, we first summarize all the variables, and then present the results of the model and the affect each variable has on time out-of-home.

### Variable Summary

Participants were followed for a full year. Occasional outages in the sensor platform and overnight travel away from home (excluded from the dataset) resulted in an average of 227 days of valid time out-of-home data for each subject. This represents 463,296 hours of continuous activity monitoring. Participants spent 4.2 ± 2.7 hours outside the home each day on average, which is consistent with previous studies on time out-of-home in older adults [3]. The average age was 86.2 ± 6.9 years; 85% of the cohort was female and 84% was white. The population had an average of 15 ± 2.5 years of education. Two participants did not complete high school; 18% completed at least some college. Because our recruitment targeted retirement communities, 72% of the cohort lived in a retirement community for older adults.

The majority of participants were cognitively intact and physically healthy. Only 10 participants had CDR = 0.5. The mean clinical walking speed was 72 ± 20.6 cm/s. The average level of pain reported on any given week was 1.85 ± 2.0 (0 is lowest, 10 is highest). Only one individual ever reported the maximum level of pain of 10, which was reported after a vertebral compression fracture.

The average Loneliness score was 34.5 ± 7.7, which is consistent with previous results using the UCLA Loneliness Scale in an elderly cohort [24]. Over half (*n =* 43) of the participants did not have a loneliness score. Seven of these individuals declined to take the survey outright and a further 7 participants selected ‘do not wish to answer’ on at least one survey question and are therefore missing a loneliness score. The remaining 29 participants with a missing loneliness score had no response on the survey whatsoever. As noted, we did not exclude any subjects with a missing loneliness score from the model, but instead modeled the missing data mechanism directly by including an indicator variable for those who did not complete the UCLA Loneliness Scale, and another for those who did not complete the loneliness scale and had a CDR score ≥ 0.5. Generally, participants reported that they were not blue for at least three days in the previous week: of the 21,364 days of data in the final dataset, only 448 of these days were marked as ‘blue’, representing positive answers from 37 subjects.

The weather and season data included in this model was specifically calculated for the Portland, OR, USA metropolitan area. On the shortest day of the year, the sun sets at 4:45 pm, over four hours earlier than it sets on the longest day of the year at 9:04 pm. The climate in Portland is relatively mild and rainy, with an average daily rainfall of 0.13 inches during the year of the study.

### Model Results

We hypothesized that cognitive function, physical ability and emotional state would all impact the time out-of-home. Our results summarized in Table 2 support this hypothesis, showing that cognitive function, physical ability and emotional state all affect the time out-of-home. We also show several of the contextual variables, including season, weather and day of the week, significantly impact time out-of-home. We next present results of the cognitive function, physical ability and emotional state variables, and then the contextual variables found to affect time out-of-home.

**Table 2:** Results of the longitudinal *tobit* panel model of time out-of-home (in hours per day) with 1000-fold bootstrap replicates for confidence intervals.

Variable	*β*	T_out_|Out	P(out)	Standard Error	z	95% CI	
Cognitive Status							
CDR	-1.687***	-1.557	0.867	0.150	-12.82	-2.220	-1.631
Physical Ability							
Pain Level	-0.123***	-0.099	0.992	0.017	-7.31	-0.156	-0.090
Clinical Walking Speed	-0.008**	-0.006	0.999	0.003	-2.64	-0.013	-0.002
Normalized Daily Walking Speed	0.115***	0.093	1.008	0.021	5.58	0.075	0.156
Emotional State							
Loneliness	-0.046***	-0.037	0.997	0.004	-11.96	-0.053	-0.038
Low Mood	-0.520***	-0.420	0.964	0.114	-4.55	-0.744	-0.296
Contextual Variables							
Age (years)	-0.046***	-0.037	0.997	0.005	-10.2	-0.055	-0.037
Sex (Female)	-1.388***	-1.122	0.904	0.070	-19.96	-1.524	-1.252
Education	0.182***	0.147	1.013	0.010	18.01	0.162	0.202
Race (White):							
Black	-0.947***	-0.766	0.935	0.101	-9.35	-1.146	-0.749
Asian	0.661***	0.535	1.046	0.094	7.02	0.477	0.846
Retirement Community	0.331***	0.267	1.023	0.076	4.34	0.181	0.480
Sunset (hour)	0.040**	0.032	1.003	0.014	2.88	0.013	0.067
Max Temperature	-0.001	0.000	1.000	0.003	-0.18	-0.006	0.005
Wind Speed	-0.014*	-0.012	0.999	0.006	-2.4	-0.026	-0.003
Precipitation	-0.201**	-0.162	0.986	0.063	-3.19	-0.324	-0.078
Weekday (Sunday)							
Monday	-0.020	-0.016	0.999	0.062	-0.32	-0.142	0.102
Tuesday	0.414***	0.335	1.029	0.060	6.85	0.295	0.532
Wednesday	0.538***	0.435	1.037	0.064	8.36	0.412	0.664
Thursday	0.589***	0.476	1.041	0.062	9.44	0.466	0.711
Friday	0.374***	0.302	1.026	0.063	5.95	0.251	0.497
Saturday	0.184**	0.149	1.013	0.066	2.8	0.055	0.313
Model Variables							
Time	4.62E-4	0.0004	1.000	2.29E-4	2.02	1.37E-5	9.10E-4
Constant	8.085***	6.536	1.557	0.547	14.79	7.014	9.156

*p<0.05;

**p<0.01;

***p<0.001

Cognitive status as assessed with the CDR scale was significantly associated with time out-of-home: those with a CDR score of 0.5 (indicating Mild Cognitive Impairment) spent an average of 1.67 hours more inside the home than those with no cognitive impairment. In addition, these individuals were 12% less likely to leave the home at all on any given day as compared to those with CDR 0.

We measured physical ability or capacity using three variables: pain level, clinical walking speed (a measure of walking speed taken annually in a clinical setting), and normalized daily walking speed (a daily measure of median walking speed in the home). Each of these variables was significantly associated with time out-of-home. Self-report of pain was negatively related to time out-of-home: a unit increase in pain level caused an average decrease in time out-of-home of 5.9 minutes each day they left the home such that participants spent 1 hour more inside the home each day they left the home during weeks where they reported the highest possible level of pain as compared to weeks where they reported the lowest possible level of pain. Individuals who walked faster in the clinic also spent slightly less time outside the home such that a unit increase in clinical walking speed of 1 cm/s caused an average decrease in time out-of-home of 37 seconds on days the participant leaves the home. Thus the participant with the slowest clinical walking speed (20 cm/s) spends one hour more outside the home on average than the individual with the fastest clinical walking speed (130 cm/s). In contrast, the participants’ normalized daily in-home walking speed was positively related to the time out-of-home each day. That is, on days where participants walked faster in the home relative to their own normal, they also spent more hours outside the home and were more likely to leave the home. However, the effect was small: increasing the normalized median daily in-home walking speed by one standard deviation caused an average increase in time out-of-home of 5 minutes.

**Fig 2:**
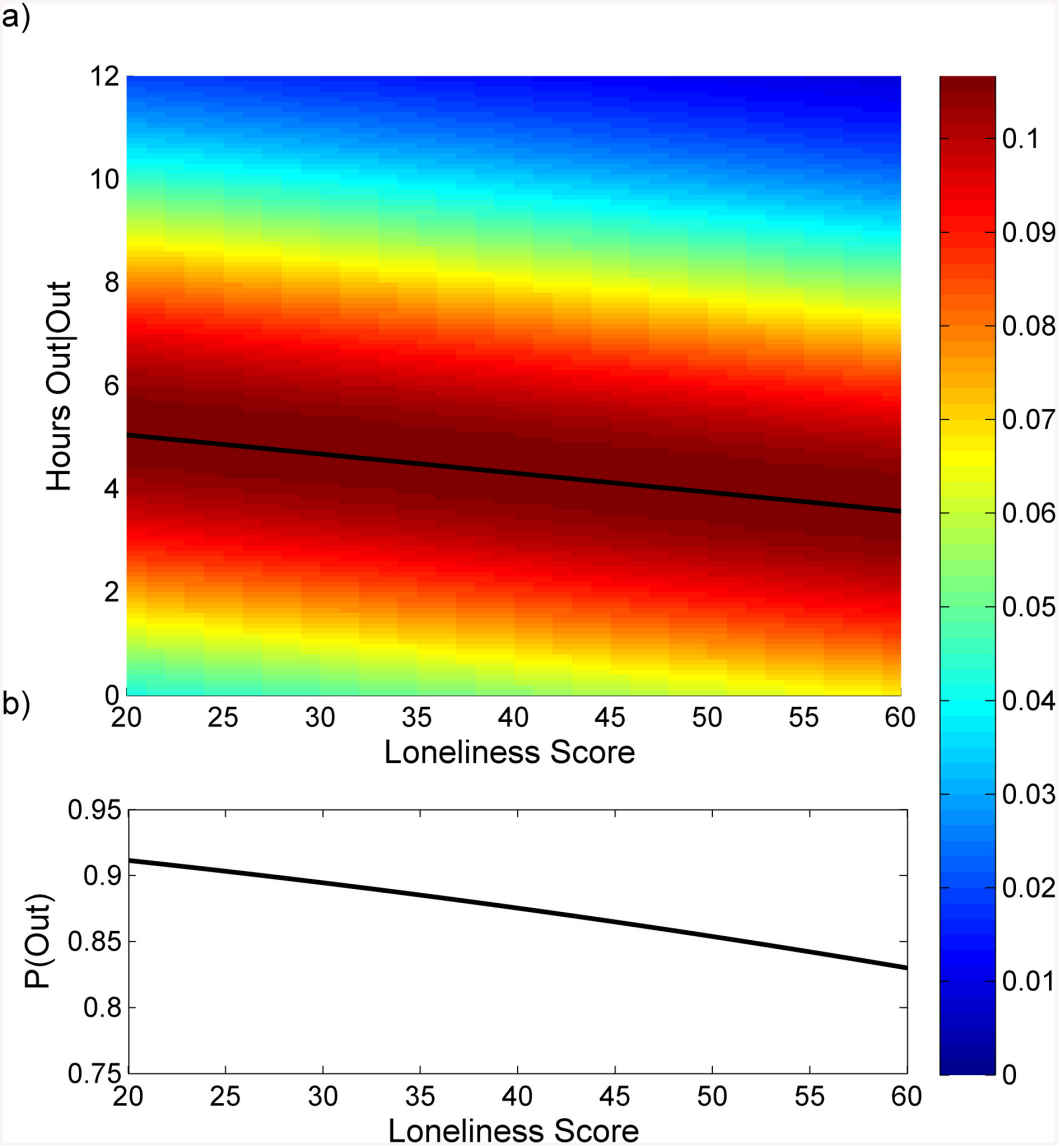
Relationship between loneliness and time out-of-home. a) Probability density of the hours spent outside the home as a function of the UCLA Loneliness Scale. To create this graph, the probability density function is calculated at each value of the UCLA Loneliness Scale using the output from the Tobit model (all other variables are held at their mean or most prevalent value for binary variables). Color represents density, and discrete probabilities were linearly interpolated for graphical clarity. The mean function, μ (black trace) has been overlaid on the plot to show central tendency. As can be seen, the average amount of time spent outside the home given a participant leaves the home decreases from 5.0 hours at a UCLA Loneliness score of 21 (the lowest observed) to 3.7 hours at a UCLA Loneliness score of 56 (the highest observed). b) Probability of leaving the home on a given day as a function of the UCLA Loneliness score. The probability of leaving drops by about 10% across the range of loneliness scores such that the loneliest individual has an 80% probability of leaving on any given day, while the least lonely has a 90% probability of leaving on any given day.

Two variables were used to represent emotional state: the UCLA Loneliness Score and weekly self-report of low mood. Individuals who reported higher loneliness scores tended to stay home more, as shown in Fig 2 which visually displays the effect of the beta coefficient on both the time spent outside the home when the participant leaves and the probability of leaving the home. In this cohort, higher loneliness was associated with spending less time outside the home. In particular, the loneliest individual (loneliness score of 56) spent an average of 1.29 hours less outside the home each day they left the home compared with the least lonely from this cohort (loneliness score of 21). In addition, the probability of leaving the home decreased by a factor of 0.997 for each additional point on the loneliness scale, such that the loneliest individual had a 10% lower probability of leaving each day compared to the least lonely.

The participant’s self-report of low mood also negatively impacted time out-of-home. For a given participant, reporting low mood was associated with spending 25 fewer minutes outside the home *each day* the participant left the home that week as compared to weeks where that participant reported they did not feel downhearted or blue.

In addition to the demographic variables (age, sex, education, and race) which may intrinsically affect time out-of-home, we included several variables hypothesized to extrinsically affect this behavior. These include weather, season, day of week, and style of living area. Each of these variables significantly affected time out-of-home, suggesting that multiple extrinsic factors should be accounted for in models of time out-of-home in order to avoid biased inference.

## Discussion

This paper presents the first steps toward an integrated model of time out-of-home and an interpretation of the variables impacting the total daily time out-of-home. Using objective, longitudinal data to assess time out-of-home, we show that time out-of-home is influenced by cognitive status, physical ability and emotional state.

Time out-of-home was significantly associated with cognitive status, likely because leaving the home requires significant cognitive resources for way finding, posture, gait, and navigation of difficult terrain. This result is consistent with other research on the effect of cognition on out-of-home behavior [34], although studies using gross summary measures of cognition such as the MMSE have found conflicting results [3]. It is important to note that in this study, only 10 participants had a CDR score ≥ 0.5, and all but one of these participants lived in a retirement community where meals could be taken at an on-site dining venue. Because living in a retirement community significantly *increases* the time out-of-home (the opposite effect of cognitive impairment), omitting the living environment from studies on time out-of-home may mask the effect of cognition on this important health behavior.

In general, physical ability positively affected the total daily time out-of-home. Both increased pain and decreased in-home walking speed, a daily measure of physical ability, were associated less with time out-of-home, although the effect of both was small. The effect of these variables may be small because of their varying potential to affect physical function over time. For example, while pain is frequently limiting, some conditions may restrict the ability to leave the home while not causing high levels of pain. The opposite may also hold: some individuals may accept or “power through” their pain for therapeutic reasons. In general, our population is not characterized by high levels of chronic pain. However, individuals with osteoarthritic pain may engage in physical activity as a treatment for pain and thus would not appear to limit their activity dramatically. We did not evaluate the specific causes of pain relative to physical activity in this study. Future studies may investigate the relationship between time out-of-home and specific causes of pain, and other more direct measures of physical function. While both higher pain levels and slower in-home walking speeds were associated with less time out-of-home, a slower clinical walking speed was related to *more* time out-of-home such that individuals who walk faster at their clinical evaluation spend less time outside the home. This counterintuitive result may indicate that walking speed in the clinic is not a good representation of daily physical ability. While subjects are asked to walk at a normal or “usual” rate during the observed timed walk, individuals experiencing difficulties walking may exhibit performance biases such that their walking speed in the clinic is dramatically faster than their regular, unsupervised walking speed at home [35]. This would explain why increased in-home walking speed increases time out-of-home, while increased clinical walking speed attenuates time out-of-home. It may also be that those who walk more slowly in the clinic spend more time outside the home for negative reasons such as doctor appointments or emergency room visits. Finally, it may be that clinically measured walking speed is not an adequate measure of walking speed over the entire year as events—such as a fall or stroke—which may dramatically decrease walking speed are not accounted for in this study [36].

Our results supported the hypothesis that time out-of-home is related to emotional state: across individuals, higher loneliness was associated with less time spent outside the home, and within the same individual reporting low mood was associated with a slight decrease in the daily time spent outside the home. This is consistent with prior research on emotional state and behavior: low mood was recently found to negatively affect time out-of-home [37], and loneliness has previously been found to impair sleep quality leading to daytime dysfunction [38] and more time inside the home [6]. Loneliness may also be associated with a smaller social network, or dissatisfaction with a larger social network which would also decrease the opportunity to leave [39], resulting in less time outside the home. Still, the effect found between low mood and time out-of-home was relatively small: only 25 minutes per day. This may be because the exact days a participant felt blue could not be determined and instead all seven days leading up to the completion of the survey were marked as ‘low mood’ even though the participant reporting low mood could have felt blue on a minimum of three days. Future studies on time out-of-home and mood should determine the days where a participant feels downhearted more precisely.

We also uncovered several contextual variables that should be accounted for when studying out-of-home behavior. First, we discovered that style of living situation may affect behavior as living in a retirement community affects time out-of-home. This may be because the opportunity to leave is higher in retirement communities: a common dining area makes it easy go out for meals on a regular basis. Many retirement communities also have community rooms where they host games and other activities for the residents. The proximity of resources has previously been shown to affect time out-of-home [18] as it may increase the opportunity space, emphasizing the need to include living environment in behavioral models. While our measure of living environment was relatively crude (comparing retirement community to independent housing), more detailed measures of proximity of resources, ease of transportation, or neighborhood demographics may also be important. This is especially true for older adults where the environment may make a larger difference. Future studies should incorporate more detailed measures of environment into models of out-of-home behavior.

Season and weather were also found to have a significant effect on time out-of-home in this cohort, although to a lesser extent than found in previous studies. One previous study investigating the relationship between season and time out-of-home found individuals in the continental US spend 73 minutes more outside the home on average on days during the summer as compared to days during the winter [13]. We found a smaller seasonal affect possibly because we accounted for more covariates and looked longitudinally for each individual, rather than taking cross-sectional snapshots across people. It may also be due to the geographical homogeneity of our cohort: we only recruited individuals from the Portland, OR, USA metropolitan area where the winters are relatively mild. Still, the results emphasize the fact that both weather and season should be included in models of human behavior, especially as both are known to also affect other behaviors such as cognitive and psychological function. For example, the amount of sunshine has previously been shown to affect mood—especially negative affect and tiredness [16] Other recent studies have linked weather with mobility [40] and pain [15].

This study was designed to overcome the limitations of prior studies on time out-of-home; still, there are limitations with our study. While we included many variables that may affect time out-of-home, it is likely that some behavioral or contextual variables were overlooked or unmeasured which may influence the results presented here. In addition, most of the participants in this cohort were in good health, cognitively intact, and relatively affluent. The results found here may not generalize to low income populations or to participants with more severe cognitive impairment or physical limitations. Future studies should investigate these relationships in more diverse populations—including diverse cultural and geographical populations. This study was able to uncover relationships between season and behavior as a result of monitoring for a full year. Nevertheless, any behavioral patterns occurring on time scales longer than one year were not captured. Future studies using this technology should incorporate additional years of data to capture such behavioral trends, as well as longer-term change in time out-of-home. In addition, this work only looked at total time inside or outside the home. It did not study either the places visited outside the home or the number of trips taken. Studies utilizing the strengths of GPS and location monitoring, which provide information on places visited, may help to further uncover key relationships between health and behavior. Total number and duration of trips during the day may also be an important health indicator, although this was outside the scope of this study. Finally, studying the timing of outings may provide further insight into the nature or objective of the outings, and thus may help more closely relate the behavior to a given health state. We also note that while the sensitivity and specificity of the classifier used to measure daily time out-of-home were high (94% and 98%, respectively), it is possible that home-specific measurement errors exist that could confound the results presented here.

In conclusion, this work presents results on the relationship between time out-of-home and cognitive status, physical ability and emotional state using objective, longitudinal in-home data. Because of the unobtrusive nature of the data capture, we were able to monitor participants daily for up to one year, providing new insights into long-term relationships between behavior and its effectors. While future studies are required to further understand these and other relationships, the approach offers a framework for testing the effectiveness of interventions while also elucidating basic relationships between health and behavior.
